# Comprehensive analyses of correlation and survival reveal informative lncRNA prognostic signatures in colon cancer

**DOI:** 10.1186/s12957-021-02196-4

**Published:** 2021-04-09

**Authors:** Meihong Gao, Yang Guo, Yifu Xiao, Xuequn Shang

**Affiliations:** School of Computer Science and Engineering, Northwestern Polytechnical University, Xi’an, People’s Republic of China

**Keywords:** Long non-coding RNAs, Secondary structure information, Regression analysis, Regulatory patterns, Risk prognosis model, Overall survival

## Abstract

**Background:**

Colon cancer is a commonly worldwide cancer with high morbidity and mortality. Long non-coding RNAs (lncRNAs) are involved in many biological processes and are closely related to the occurrence of colon cancer. Identification of the prognostic signatures of lncRNAs in colon cancer has great significance for its treatment.

**Methods:**

We first identified the colon cancer-related mRNAs and lncRNAs according to the differential analysis methods using the expression data in TCGA. Then, we performed correlation analysis between the identified mRNAs and lncRNAs by integrating their expression values and secondary structure information to estimate the co-regulatory relationships between the cancer-related mRNAs and lncRNAs. Besides, the competing endogenous RNA regulation network based on co-regulatory relationships was constructed to reveal cancer-related regulatory patterns. Meanwhile, we used traditional regression analysis (univariate Cox analysis, random survival forest analysis, and lasso regression analysis) to screen the cancer-related lncRNAs. Finally, by combining the identified colon cancer-related lncRNAs according to the above analyses, we constructed a risk prognosis model for colon cancer through multivariate Cox analysis and also validated the model in the colon cancer dataset in TCGA cohorts.

**Results:**

Six lncRNAs were found highly correlated with the overall survival of colon cancer patients, and a risk prognosis model based on them was constructed to predict the overall survival of colon cancer patients. In particular, EVX1-AS, ZNF667-AS1, CTC-428G20.6, and CTC-297N7.9 were first reported to be related to colon cancer by using our model, among which EVX1-AS and ZNF667-AS1 have been predicted to be related to colon cancer in LncRNADisease database.

**Conclusions:**

This study identified the potential regulatory relationships between lncRNAs and mRNAs by integrating their expression values and secondary structure information and presented a significant 6-lncRNA risk prognosis model to predict the overall survival of colon cancer patients.

**Supplementary Information:**

The online version contains supplementary material available at (10.1186/s12957-021-02196-4).

## Background

Colon cancer is a common cancer with high incidence and mortality worldwide [1, 2]. It can be divided into different subtypes according to clinical molecular characteristics [3]. The occurrence of colon cancer is closely related to many factors, such as age, lifestyle, diet, environmental pollution, and disease history [4]. Some genes have been found to be involved in the occurrence of colon cancer. For example, KRAS proto-oncogene and TP53 tumor suppressor gene are related to the development and prognosis of colon cancer [5, 6]. Likewise, INHBA plays an immunomodulatory role in colon cancer [7], and BRIP1 is related to the susceptibility of colon cancer [8]. At present, although radical resection combined with chemotherapy can improve the survival rate of colon cancer, the treatment results are still unsatisfactory [9]. Therefore, it is important to identify causal regulators at the genome level for understanding the basic mechanism of cancer occurrence, thus to improve the precision of cancer treatments. In recent years, numerous studies have shown that there are some potential relationships between the abnormal expression of long non-coding RNA (lncRNA) and the occurrence of cancer [10–14]. The detection of cancer-associated lncRNA has proven to be a particularly valuable method for effective cancer diagnosis [15, 16]. Because lncRNA can specifically bind to mRNA/miRNA and cause their abnormal expression, it can be used as a promising target for the diagnosis and treatment of colon cancer [17]. To this end, it is necessary to reveal the regulatory mechanism of lncRNAs in colon cancer and develop new therapies for human colon cancer.

Long noncoding RNA is defined as a transcript longer than 200 nucleotides [18]. Comparing with mRNA and other non-coding RNAs, lncRNA has relatively low conservation and low expression levels [19]. This is because its sequence has a higher mutation rate than mRNA and other non-coding RNAs during evolution, and it does not have to participate in the translation process. Recently, more and more lncRNAs have been identified, and 14826 lncRNAs have been annotated by the GENCODE (https://www.gencodegenes.org/) consortium (v22). Many studies have shown that lncRNAs are involved in some major regulatory processes and are closely related to the occurrence of cancer [13, 14, 20–23]. Identifying lncRNAs related to human diseases can help to understand the mechanisms of human disease at the lncRNA level. On the one hand, the secondary structure of lncRNA can provide useful information for inferring the regulatory relationships in the occurrence of human diseases [24]. On the other hand, lncRNA is considered to be an important part of the competing endogenous RNA (ceRNA) regulatory network, and the construction of lncRNA-related ceRNA regulatory relationships helps to understand the mechanism of lncRNA in colon cancer [25, 26]. Currently, several lncRNAs, such as HOTAIR, HOXB-AS3, UCA1, and MALAT1, have been found to be related to the occurrence of colon cancer [27–30].

Understanding the regulatory mechanism of lncRNA in the occurrence and development of colon cancer can provide informative prognostic signatures for patients with poor prognosis [10, 15]. Although experimental methods can identify lncRNAs associated with colon cancer, they are time-consuming and costly. For example, CEL-seq2 costs $2420 when sequencing 110 cells at a depth of 1 million reads [31], Drop-seq costs $1110 when sequencing 254 cells at a depth of 1 million reads [31], and MARS-seq costs 1380$ when sequencing 160 cells at a depth of 1 million reads [31]. Moreover, it takes several days to generate sequencing libraries and sequencing data. Therefore, it is essential to develop computational methods to identify lncRNAs associated with colon cancer. Many studies have been performed to use lncRNA signatures to estimate the samples’ survival time (based on overall survival) of colon cancer [32–35] and other cancers (gastric cancer [36], clear cell renal cell carcinoma [37], and breast cancer [38]) through computational methods. These methods have been proven to have good prognostic performance on their own data sets, but they have a common limitation that they only considered the expression information of lncRNA and ignored the important role of lncRNA secondary structure in the regulation process. Therefore, it is necessary to consider both the expression and structure information to construct an effective prognostic model.

In this study, we performed an integrative analysis of the correlation and survival of colon cancer and revealed some significant lncRNA signatures that can be used for the prognosis of colon cancer. Specifically, a risk prognostic model based on the identified lncRNA signatures was constructed and verified, which not only can help to understand the mechanism of colon cancer at the long non-coding RNA level but also provide the promising lncRNA signatures candidates for the diagnosis of colon cancer. The contributions of this study can be summarized as follows. (1) We predicted the regulatory relationships between lncRNAs and mRNAs by integrating their expression values and secondary structure information. (2) Two new lncRNAs (CTC-428G20.6 and CTC-297N7.9) related to colon cancer were discovered. (3) A significant six-lncRNA (RP11-798K3.2, RP11-400N13.2, EVX1-AS, CTC-428G20.6, ZNF667-AS1, and CTC-297N7.9) risk prognosis model was presented to estimate the overall survival of colon cancer patients. Among these six lncRNAs, EVX1-AS and ZNF667-AS1 have been predicted to be related to colon cancer in LncRNADisease V2.0 (http://www.rnanut.net/lncrnadisease/) (the latter was verified in the correlation analysis); RP11-798K3.2 and RP11-400N13.2 have been proven to be related to colon cancer by previous studies [34, 35].

## Methods

**Fig. 1:**
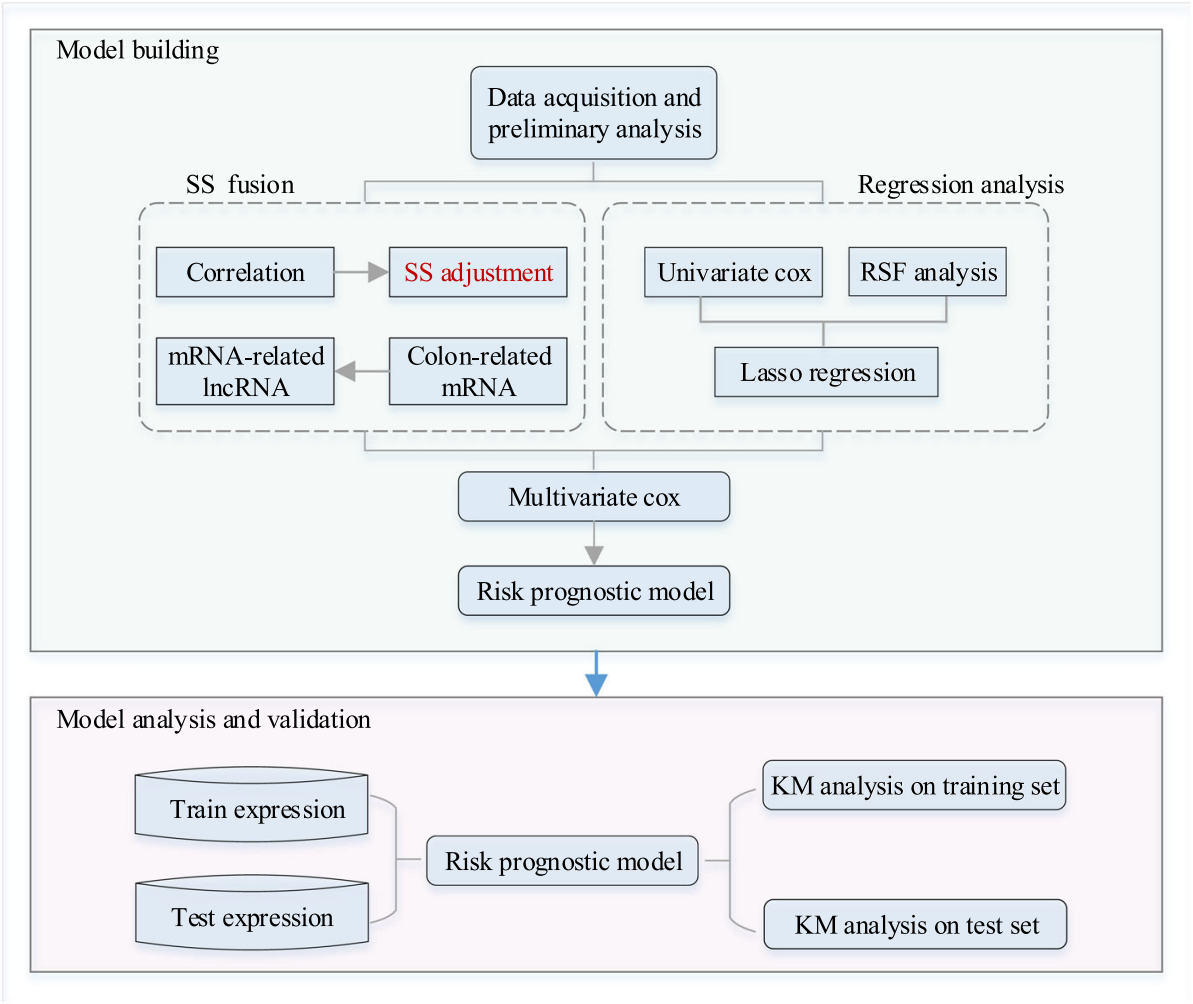
The flow chart of the analysis process. Secondary structure (SS) fusion refers to the combination of expression value correlation and secondary structure correlation. Regression analysis is a combination of univariate Cox analysis, random survival forest (RSF) analysis, and lasso regression analysis. Kaplan-Meier (KM) analysis refers to constructing the KM survival curve based on the risk prognosis model

The workflow of our study is shown in Fig. 1. There are two modules in the framework, the first is the construction of the prognostic model, and the second is the analysis and validation of the model.

### Data acquisition and preliminary analysis

The original RNA-seq expression data and clinical information (race, ethnicity, vital status, days to death, age at index, year of diagnosis, tumor stage, days to last follow up, etc.) of colon adenocarcinoma (COAD) were downloaded from TCGA database (https://portal.gdc.cancer.gov/) by using GDC Data Transfer Tool, which contained 451 tumor samples and 41 adjacent normal samples. Among these samples, 447 had complete clinical information. After excluding samples with too short overall survival (less than 10 days), 411 were left (See Supplementary Table S1, Additional File 1). The expression profiles of lncRNA and mRNA of colon cancer were obtained through the annotation file of the GENCODE (v22: determined by the annotation information used in TCGA) database. Finally, there were 14826 annotated lncRNAs and 19814 annotated mRNAs for subsequent analysis.

To discover the lncRNAs and mRNAs related to colon cancer, we conducted a preliminary differential analysis on the expression profiles of colon cancer. The expression profiles of lncRNAs and mRNAs were normalized before performing differential expression analysis by using the edger package (https://bioconductor.org/packages/release/bioc/html/edgeR.html) of R software. The normalization method used was the trimmed mean of *M* value (TMM). Specifically, the expression profiles were divided into colon cancer and control group, and the limma package [39] of R software was used to find out the differentially expressed RNAs (lncRNAs and mRNAs) between colon cancer and adjacent tissues. The expression differences were evaluated by the fold change (represent the range of changes from initial to final values) and the related adjusted *p* values. The *p* values of lncRNAs and mRNAs were obtained by *t* test and corrected by Benjamini-Hochberg (BH) [40]. Differentially expressed lncRNAs and mRNAs were acquired by setting the adjusted *p* value <0.01 and the absolute value of logFC >1.5. The up/downregulation mRNAs and lncRNAs were identified for subsequent co-expression analysis.

### Co-expression analysis and secondary structure information fusion

Co-expression analysis can be used to predict the correlation between mRNA and lncRNA at the expression level. By analyzing the correlation coefficient, we can find the degree of correlation between lncRNA and mRNA. Practically, a co-expression matrix $C= \left (\begin {array}{ll} C_{LL}&C_{LM}\\ C_{ML}&C_{MM} \end {array}\right)$ was acquired by using the cor method of the stats package in R software. *C*_*LL*_ is the Spearman correlation matrix between lncRNAs; *C*_*LM*_ is the Spearman correlation matrix between lncRNAs and mRNAs; *C*_*ML*_ is the Spearman correlation matrix between mRNAs and lncRNAs; *C*_*MM*_ is the Spearman correlation matrix between mRNAs. Obviously, *C*_*LM*_ is equal to $C_{ML}^{\mathrm {T}}$. Suppose that *C*(*m*,*l*) is an element in the *C*_*ML*_ matrix, which represents the Spearman’s rank correlation between mRNA *m* and lncRNA *l*. Assuming there are *p* mRNAs and *q* lncRNAs, the Spearman’s rank correlation coefficient [41] between the *m*th mRNA and the *l*th lncRNA is defined as follows:

1 $$ C(m,l)=1-\frac{6\sum d_{i}^{2}}{samp\_{no}(samp\_{no}^{2}-1)}  $$

where *d*_*i*_ represents the difference between the rank of *m* and *l*, and *samp*_*no*_ is the number of colon cancer samples. *C*(*m*,*l*) ranges from − 1 to 1, and the greater the absolute value of *C*(*m*,*l*), the stronger the correlation between mRNA *m* and lncRNA *l*. A correlation matrix with *p* rows and *q* columns was obtained by setting the threshold of the correlation coefficient to a specific threshold *α* from 0 to 1:

2 $$ C_{ML}(\alpha)= \left[\begin{array}{ccc} C(1,1)&\cdots&C(1,q)\\ \vdots&\ddots&\vdots\\ C(p,1)&\cdots&C(p,q) \end{array}\right]  $$

where *p* denotes the number of mRNAs in the co-expression relationship, and *q* denotes the number of lncRNAs in the co-expression relationship. In general, we suppose that the correlation is weak when *α*<0.3; the correlation is sensible when 0.3≤*α*≥0.7; the correlation is stronger when *α*>0.7. In each row and column of the matrix *C*_*ML*_(*α*), at least one number has an absolute value greater than or equal to *α*. *N*_*r*_(*i*) is the number of *C*(*m*,*l*)≥*α* in the *i*th rows, *N*_*c*_(*j*) is the number of *C*(*m*,*l*)≥*α* in the *j*th columns, where *N*_*r*_(*i*)∈{1,⋯,*q*} and *N*_*c*_(*j*)∈{1,⋯,*p*}.

In addition, in order to find the intrinsic and potential regulatory relationship between lncRNA and mRNA, we also consider the secondary structure information of lncRNA and mRNA to estimate the correlation between them at the sequence structure level. We define the correlation coefficient between mRNA *m* and lncRNA *l* on the secondary structure as:

3 $$ E(m,l)=\frac{\sum_{s=1}^{u(m)}\sum_{t=1}^{v(l)}\frac{MFE_{st}}{LEN\_M_{s}+LEN\_L_{t}}}{u(m) \cdot v(l)}  $$

where *E*(*m*,*l*) denotes the secondary structure correlation of mRNA *m* and lncRNA *l*, *MFE*_*rs*_ denotes the minimum free energy (the minimum energy required to make the RNA molecule have a stable secondary structure [42]) of concatenation sequence of the transcript *s* of mRNA *m* and the transcript *t* of lncRNA *l*. *MFE*_*st*_ was calculated by RNAcofold [43]. In formula (3), *u*(*m*) denotes the number of transcripts of mRNA *m*, *v*(*l*) denotes the number of transcripts of lncRNA *l*, *LEN*_*M*_*r*_ denotes the length of the transcript *r* of mRNA *m*, and *LEN*_*L*_*s*_ denotes the length of the transcript *s* of lncRNA *l*. For each *E*(*m*,*l*) in matrix *E*_*ML*_(*α*), a corresponding *E*^′^(*m*,*l*) is defined as:

4 $$ E'(m,l)=\frac{E(m,l)-\min{E_{ML}(\alpha)}}{\max{E_{ML}(\alpha)}-\min{E_{ML}(\alpha)}}  $$

The secondary structure correlation matrix *E*_*ML*_(*α*) corresponding to the Spearman’s rank correlation matrix *C*_*ML*_(*α*) was obtained through *E*(*m*,*l*). After matrix *E*_*ML*_(*α*) was min-max normalized, matrix $E^{\prime }_{ML}(\alpha)$ was normalized to the range [0,1]. The Spearman correlation matrix and the secondary structure correlation matrix were fused to obtain an adjusted correlation matrix composed of differentially expressed lncRNAs and mRNAs. The adjusted correlation matrix *AC*_*ML*_(*α*) is defined as:

5 $$ AC_{ML}(\alpha)= \left[\begin{array}{ccc} AC(1,1)&\cdots&AC(1,q)\\ \vdots&\ddots&\vdots\\ AC(p,1)&\cdots&AC(p,q) \end{array}\right]  $$

where *p* and *q* denote the number of mRNAs and lncRNAs, respectively. Each *AC*(*m*,*l*) in Matrix *AC*_*ML*_(*α*) is defined as:

6 $$ {}AC(m,l)=\left\{ \begin{array}{r} \max(\vert C(m,l)\vert, E'(m,l)), C(m,l) \ge 0\\ -\max(\vert C(m,l)\vert, E'(m,l)), C(m,l) < 0 \end{array} \right.  $$

where *AC*(*m*,*l*) represents the adjusted correlation coefficient between mRNA *m* and lncRNA *l*, which was determined by *C*(*m*,*l*) and *E*^′^(*m*,*l*). *AC*(*m*,*l*) combines expression value information and secondary structure information, which can fully reflect the correlation between mRNA m and lncRNA l.

In order to further analyze the potential regulation mode of lncRNA after the secondary structure correlation fusion, we constructed a competing endogenous RNA (ceRNA) regulation network based on the adjusted co-regulation relationships. The ceRNA network plays an important regulatory role in colon cancer, and the lncRNA in it can be used as biomarkers for the prognosis of colon cancer. In the process of post-transcriptional regulation, lncRNA and mRNA compete for binding to miRNA to form a ceRNA regulatory network. In our framework, the ceRNA regulation network was constructed based on lncRNAs and mRNAs (both RNAs were differentially expressed). Firstly, mRNA-targeted miRNAs were collected from TargetScan database (http://www.targetscan.org/vert_72/). Secondly, lncRNA-targeted miRNAs were collected from miRcode database (http://www.mircode.org/). Thirdly, common miRNAs found in the above two steps were screened out. Finally, the ceRNA regulatory network was built and visualized through the interaction between mRNAs, lncRNAs, and their common miRNAs by using Cytoscape v3.6.1 [44].

Furthermore, to comprehend the potential biological effects of dysregulated mRNA related to lncRNA, function and pathway enrichment analyses were carried out by using DAVID on line tools (version 6.8, https://david.ncifcrf.gov/). Specifically, the detected mRNAs were enriched on GO (Molecular Function, Biological Process, and Cellular Component) terms and KEGG pathways respectively. Finally, the items with *p* value < 0.05 were used to interpret the functions of the detected mRNAs in colon cancer.

### Traditional regression analysis

We used the survival package [45] to perform univariate Cox analysis to detect the relationships between dysregulated lncRNAs and the overall survival of colon cancer patients (lncRNAs with log-rank *p* value <0.05 were considered significant). The random survival forest (RSF) analysis was performed to access the link between differentially expressed lncRNAs and the overall survival of colon cancer patients by using randomForestSRC package (https://cran.r-project.org/web/packages/randomForestSRC/index.html) in R software. The union of the outputs of univariate Cox analysis and RSF analysis was used for lasso regression analysis to detect cancer-related lncRNAs. Significant lncRNA signatures were obtained by selecting items with non-zero regression coefficients in the results of lasso analysis.

### Comprehensive analysis and construction of risk prognosis model

Considering the previous regression analysis may lose some lncRNA features that have no obvious relationships between expression level and survival time but may affect survival time through coordination (based on overall survival), we further developed a new method to identify those survival-related lncRNAs. In detail, we found these missing lncRNA features through the following: (a) downloaded the pathogenic mRNAs of colon cancer from the Cosmic (https://cancer.sanger.ac.uk/cosmic/) disease database, (b) identified the related pathogenic mRNAs in the co-regulatory network, and (c) identified the lncRNAs related to the pathogenic mRNAs in the co-expression network.

By combining the preliminarily identified lncRNAs (from traditional regression analysis) with the lncRNAs associated with the pathogenic mRNAs found above, multivariate Cox analysis was carried out to identify lncRNAs associated with the prognosis of colon cancer. Specifically, we tried to identify k lncRNA signatures to estimate the overall survival of colon cancer. A matrix *P*_*SL*_ containing g samples’ expression profile, overall survival, and vital status is defined as *P*_*SL*_=(*h*_1_,*h*_2_,...,*h*_*g*_). Here, *h*_*i*_ is a vector and the transposition of *h*_*i*_ is defined as $h_{i}^{\mathrm {T}}=(e_{i1},...e_{ik},v_{i},o_{i})$, where *e*_*ij*_ denotes the expression value of the *i*th sample on the *j*th lncRNA, *v*_*i*_ denotes the survival status of the *i*th sample, and *o*_*i*_ denotes the overall survival of the *i*th sample. Through the regression coefficients and expression values of k lncRNAs, the following predictive formula for colon cancer sample *i* can be obtained:

7 $$ R(i)=\sum_{j=1}^{k}\beta_{j} \cdot e_{ij}  $$

where *R*(*i*) denotes the risk score of the *i*th colon cancer sample, and *β*_*j*_ denotes the regression coefficient of the *j*th lncRNA signature. A prognosis model of colon cancer samples based on lncRNA signatures was obtained through the above formula. In particular, the model was analyzed and verified on the TCGA data set.

### Construction of Kaplan-Meier curve

We calculated the risk score of all colon cancer samples based on the risk prognostic model. The risk scores were divided into high-risk group and low-risk group by setting a specific cutoff. The risk level is obtained as follows:

8 $$ RL(i)=\left\{ \begin{array}{ll} low, & R(i) < cut\_off\\ high, & R(i) \ge cut\_off \end{array} \right.  $$

where *RL*(*i*) denotes the risk level of the *i*th sample, and the default *cutt*_*off* is the median risk score of all colon cancer samples. Then, the Kaplan-Meier (KM) survival curve based on the overall survival, vital status, and prognostic risk of the samples was constructed as follows. (1) The survival rate of high-risk samples was calculated. (2) The survival rate of low-risk samples was calculated. (3) The KM curve based on overall survival and survival rate was constructed. Specifically, the construction of the KM curve is achieved by the survival package [45] of the R software. There are two lines in the KM survival curve, one is for high-risk samples and the other is for low-risk samples. Ideally, there should be a clear difference in the survival rate of samples with high and low risks, that is, there is no obvious crossover between the two lines.

## Results

### Dysregulated lncRNAs and mRNAs

**Fig. 2:**
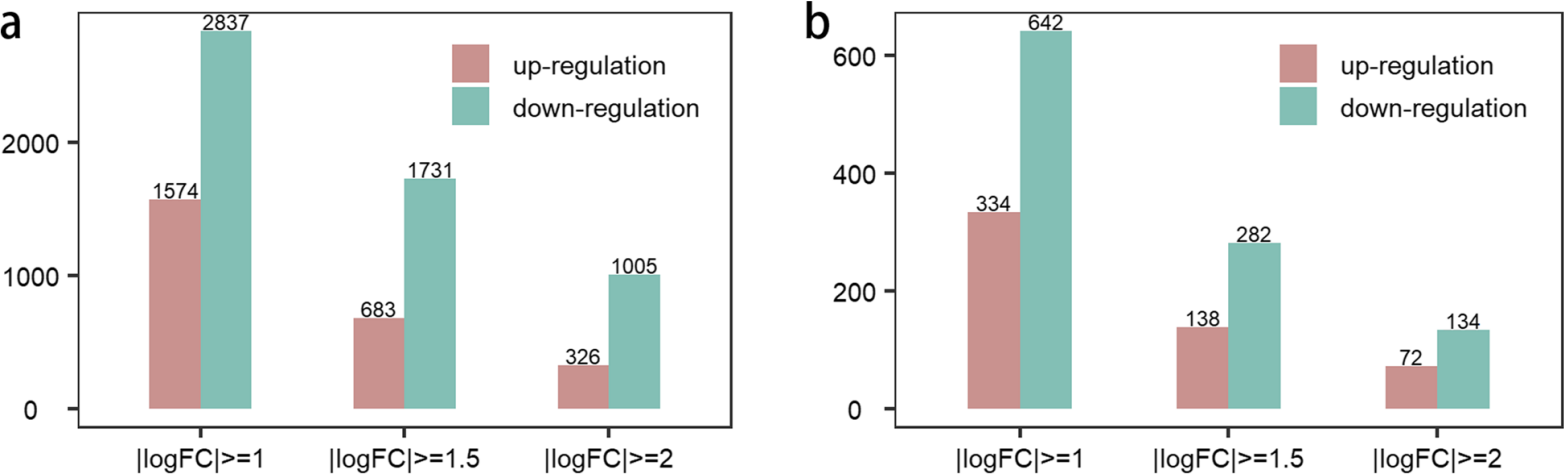
The number of up/downregulated mRNAs and lncRNAs. **a** The number of up/downregulated mRNAs (adjusted *p* value <0.01). **b** The number of up/downregulated lncRNAs (adjusted *p* value <0.01)

**Fig. 3:**
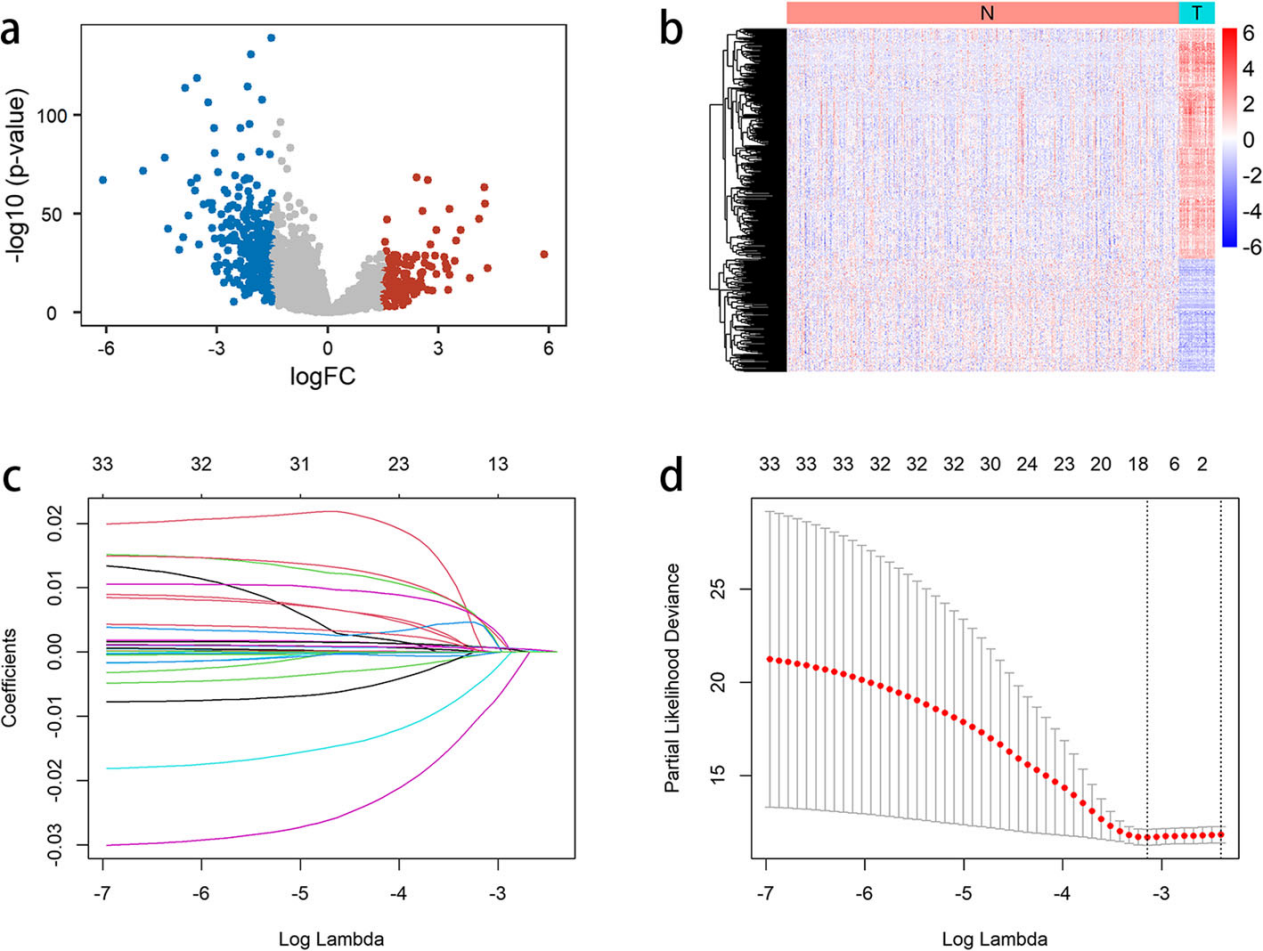
The results of difference analysis and lasso regression analysis. **a** The volcano plot of differentially expressed lncRNAs. **b** The heatmap of differentially expressed lncRNAs. **c** Lasso regression coefficients. **d** The partial likelihood deviance of lasso regression coefficients

The numbers of up/downregulated mRNAs and lncRNAs based upon three distinct thresholds of fold change are shown in Fig. 2. When the absolute value of logFC (logarithm of fold change) >= 1.5, a total of 2414 dysregulated mRNAs (683 were up-regulated and 1731 were downregulated) and 420 dysregulated lncRNAs (138 were upregulated and 282 were down-regulated) were identified. The volcano plot and heatmap of the differentially expressed lncRNAs are shown in Fig. 3a and b, respectively. It can be discovered that there is a significant dysregulation in the expression of lncRNAs in colon cancer, and the downregulation rate is greater than the upregulation rate.

### Correlation and gene function

**Fig. 4:**
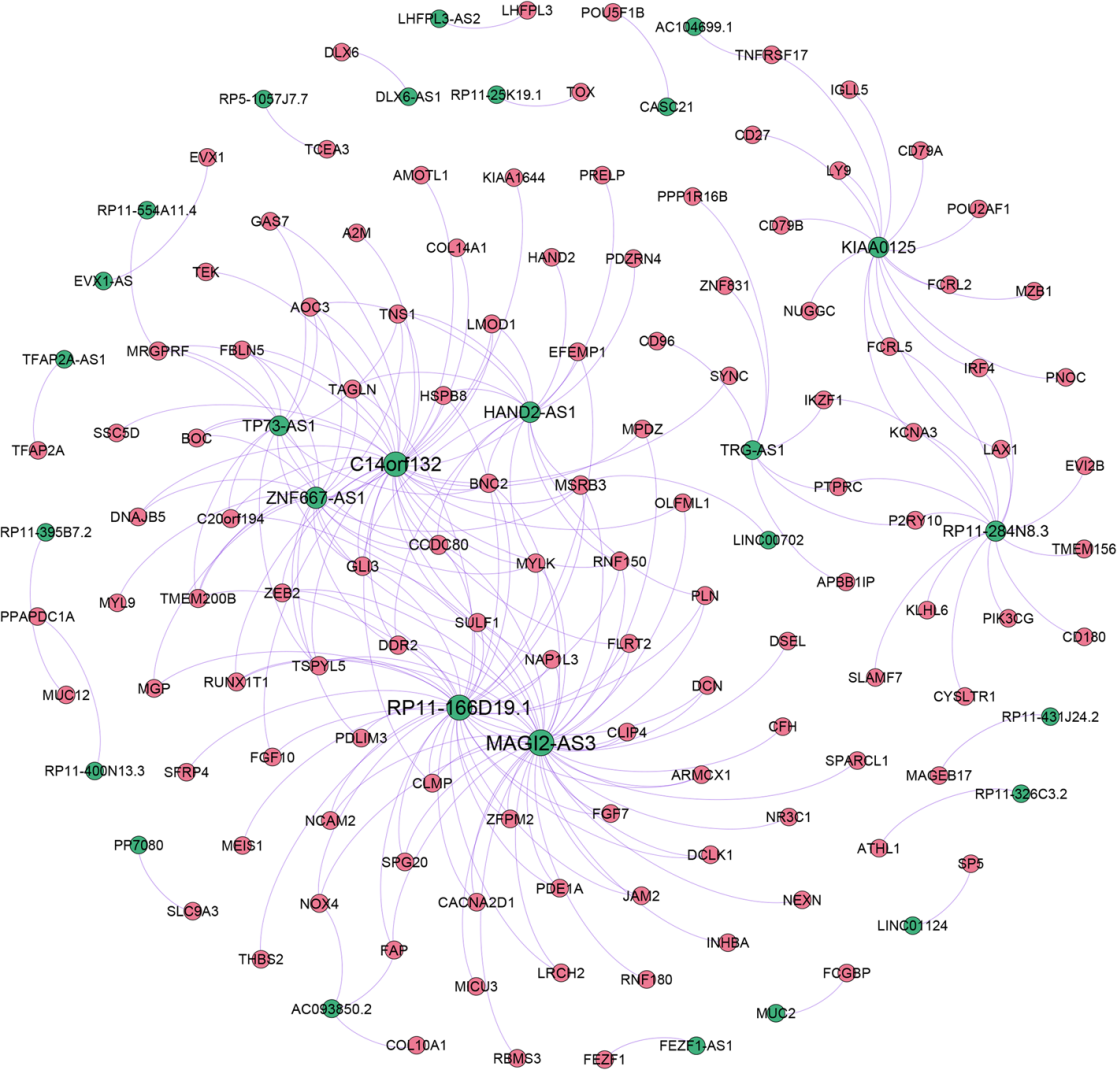
Co-regulatory network of mRNAs and lncRNAs. The red and green nodes represent mRNAs and lncRNAs, respectively. The size of the node is proportional to the degree of the node. The thickness of the edge is proportional to the strength of the correlation

In the co-expression analysis, 115 mRNA and 27 lncRNA were retained by setting *α*=0.8. This means that the order of the matrix *C*_*ML*_(0.8) was 115∗27. Then, a regulatory network based on these 115 lncRNAs and 27 mRNAs were constructed (220 interactions, Fig. 4). As shown in Fig. 4, it can be found that 9 of these 27 lncRNAs have a high degree in the regulatory network. The top-3 lncRNAs with the highest degrees are MAGI2-AS3, RP11-166D19.1, and C14orf132 (degrees are 42, 38, and 35 respectively). Actually, MAGI2-AS3 is found to promote colon cancer progression by regulating the miR-3163/TMEM106B axis [46]. There were 42 differentially expressed mRNAs related to MAGI2-AS3. The differential expression of these mRNAs may be related to the regulatory relationship between MAGI2-AS3 and miR-3163.

The correlation coefficients before and after the secondary structure correlation adjustment are shown in Table 1, Table 2 respectively (*α*=0.9). Especially, some potential correlations are discovered through secondary structure correlation adjustment. Among the 48 interaction coefficients, 11 are unchanged and 37 are adjusted through secondary structure correlation. These 37 numbers vary from 0.043878052 to 0.799352838 based on the original value.

**Table 1:** Spearman’s rank correlation (threshold=0.9)

	CASC21	FEZF1.AS1	KIAA0125	RP11.25K19.1	MAGI2.AS3	DLX6.AS1
POU5F1B	0.921046	− 0.272906	− 0.198371	− 0.335172	− 0.083741	0.207652
DDR2	− 0.055541	− 0.145469	0.295115	0.056139	0.901414	− 0.019750
FEZF1	− 0.214465	0.905106	0.078724	0.249223	− 0.129703	− 0.090882
MZB1	− 0.156008	0.047820	0.901879	0.169155	0.262776	− 0.068253
TOX	− 0.398056	0.234735	0.209269	0.915596	0.112641	0.030225
CACNA2D1	0.014141	− 0.193714	0.247710	0.047105	0.906147	0.024552
FCRL5	− 0.167879	0.054787	0.917191	0.154289	0.363034	− 0.087492
DLX6	0.219619	− 0.153076	− 0.088336	− 0.009765	− 0.046828	0.905186

**Table 2:** Adjusted correlation (threshold = 0.9)

	CASC21	FEZF1.AS1	KIAA0125	RP11.25K19.1	MAGI2.AS3	DLX6.AS1
POU5F1B	0.921046	− 0.382356	− 0.411440	− 0.335172	− 0.304581	0.296538
DDR2	− 0.366222	− 0.561156	0.613738	0.427867	0.901414	− 0.261861
FEZF1	− 0.656697	1.000000	0.819006	0.626732	− 0.598957	− 0.365746
MZB1	− 0.769841	0.847173	0.952822	0.716688	0.719770	− 0.398660
TOX	− 0.398056	0.234735	0.209269	0.915596	0.112640	0.074103
CACNA2D1	0.160565	− 0.354152	0.368323	0.246121	0.906147	0.164745
FCRL5	− 0.474429	0.557145	0.917191	0.479419	0.466437	− 0.362183
DLX6	0.381330	− 0.574835	− 0.615991	− 0.441327	− 0.387686	0.905186

**Fig. 5:**
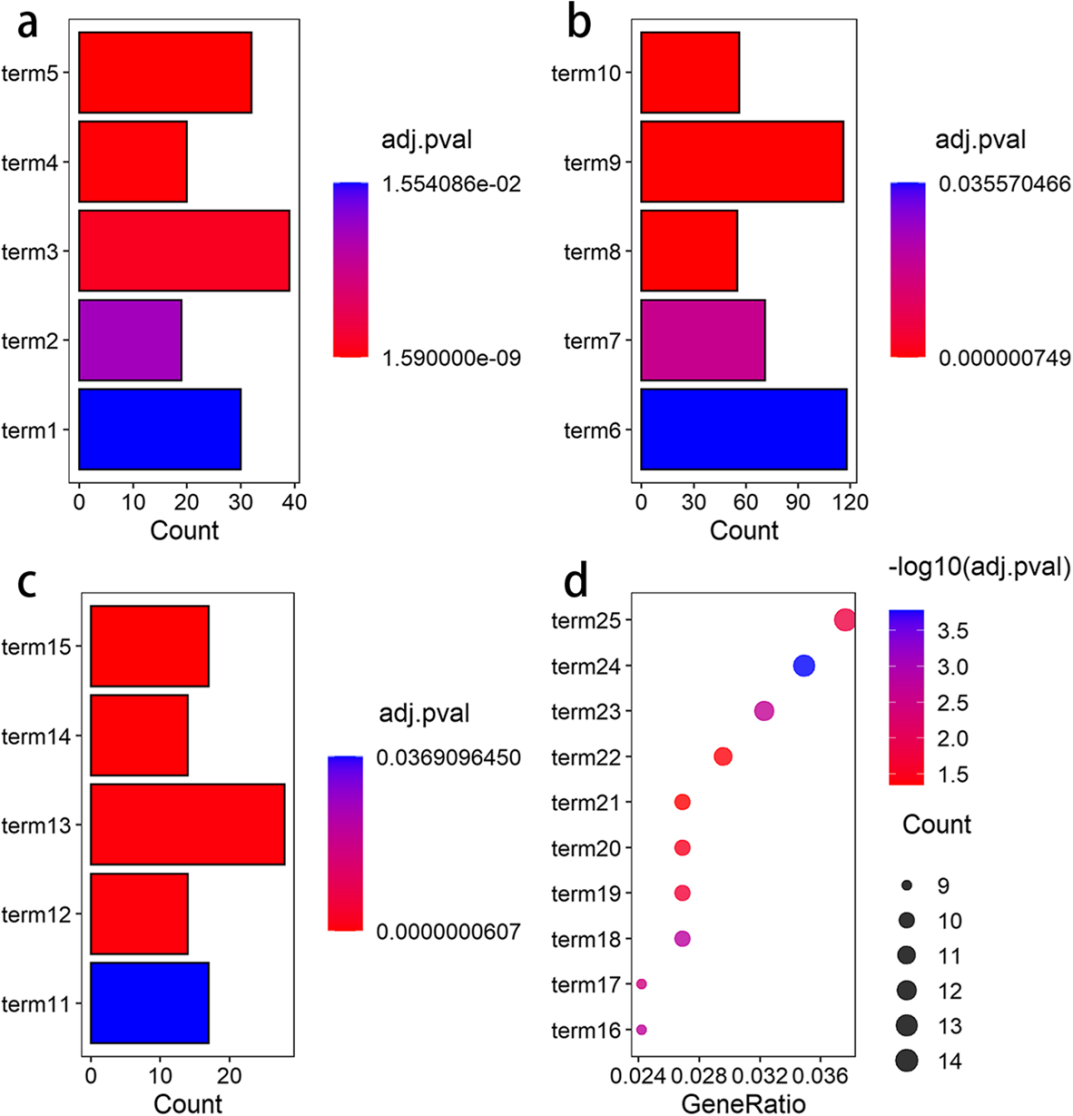
Gene Ontology (GO) terms and Kyoto Encyclopedia of Genes and Genomes (KEGG) pathway analysis. **a** GO analysis of biological process. The term1-5 represents positive regulation of transcription, transcription from RNA polymerase II promoter, signal transduction,inflammatory response, and cell adhesion, respectively. **b** GO analysis of cellular component. The term6-10 represents integral component of membrane, extracellular exosome, extracellular region, plasma membrane, and integral component of plasma membrane, respectively. **c** GO analysis of molecular function. The term11-15 represents sequence-specific DNA binding,transcriptional activator activity, calcium ion binding, receptor activity, and heparin binding, respectively. **d** KEGG pathway analysis. The term16-25 represents Vascular smooth muscle contraction, platelet activation, cell adhesion molecules (CAMs), Rap1 signaling pathway, Ras signaling pathway, cytokine-cytokine receptor interaction, neuroactive ligand-receptor, interaction focal adhesion, calcium signaling pathway, and PI3K-Akt signaling pathway, respectively

The results of GO terms and KEGG pathway enrichment analysis show that these mRNAs are related to some regulation of system processes (Fig. 5). It can be found that the target mRNAs are mainly enriched in the signal transduction of the biological process. (Fig. 5a). Disorders of signal transduction pathways in normal cells can cause cancers. As for the cellular component process, it can be found that the target mRNAs are mainly enriched in the integral component of membrane (Fig. 5b). The oligosaccharides on the cell membrane are the markers of recognition between cells. The behavior of tumor cells is related to changes in cell membrane oligosaccharides. When it comes to the molecular function process, it can be found that the target mRNAs are mainly enriched in the calcium ion binding (Fig. 5c). The calcium ions play a considerable role in the process of cell carcinogenesis, and the binding of calcium ions may be related to the occurrence of cancer. The KEGG pathways are chiefly enriched in the PI3K-Akt signaling pathway (Fig. 5d). PI3K-Akt signaling pathway is a principal intracellular signal transduction pathway, which plays a critical role in cell apoptosis and survival, and is high correlated with tumor occurrence. It has been reported that the activity of PI3K-Akt signaling pathway is increased in colon cancer [47]. The enrichment of PI3K-Akt signaling pathway makes the signals about cell survival, cell growth and cell cycle activated frequently, which leads to the occurrence of colon cancer.

### ceRNA regulatory network

**Fig. 6:**
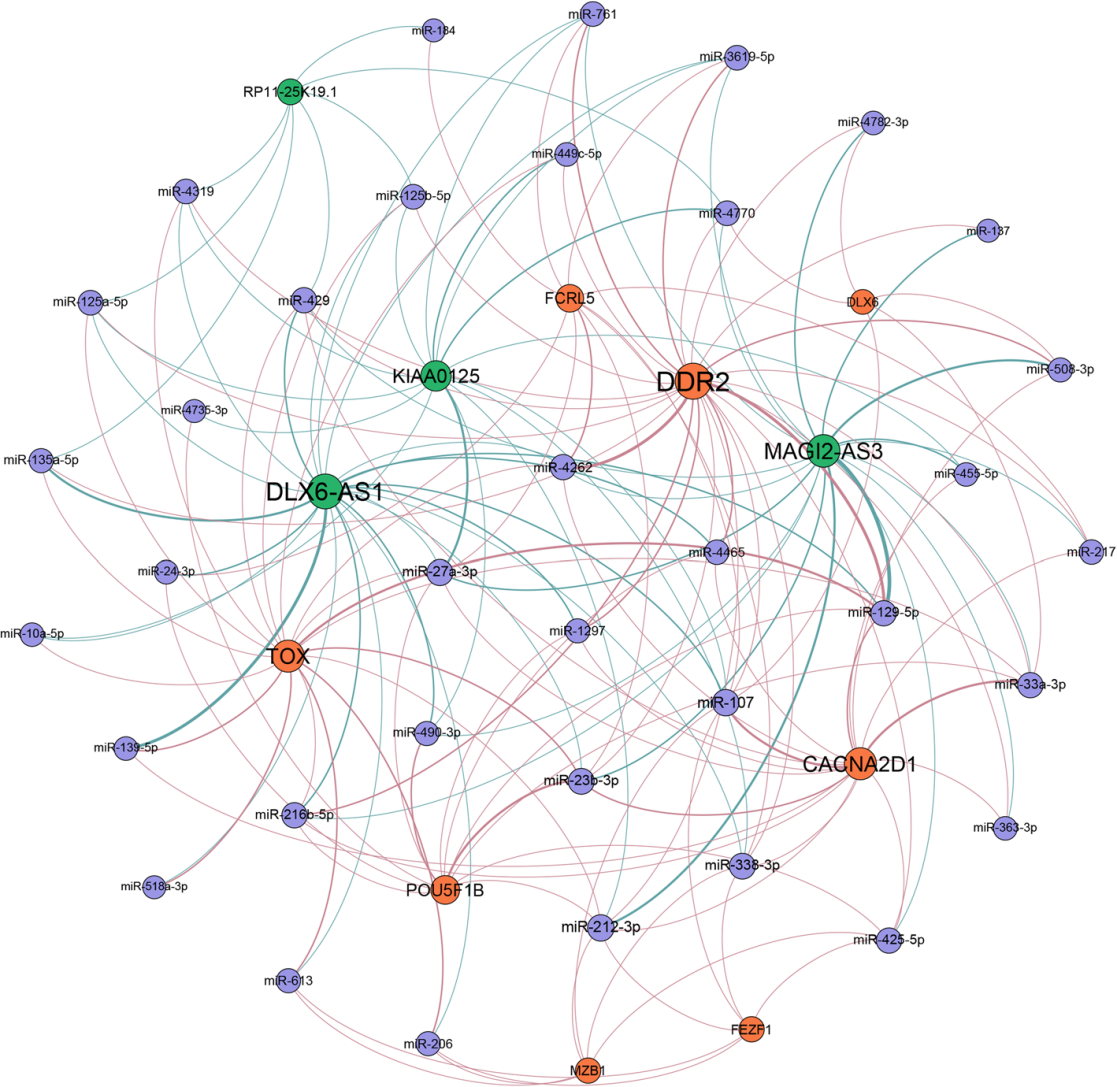
CeRNA regulatory network. The orange, green, and purple nodes represent mRNA, lncRNA, and miRNA, respectively. The orange and green edges represent mRNA-miRNA interaction and lncRNA-miRNA interaction, respectively. The size of the node is proportional to the degree of the node. The thickness of the edge is proportional to the strength of the correlation

A strongly related ceRNA network was constructed by uniting the lncRNA-miRNA interactions and the miRNA-mRNA interactions (Fig. 6). As shown in Fig. 6, there are 4 lncRNAs, 8 mRNAs, and 36 miRNAs in this ceRNA regulatory network. The degrees of lncRNA RP11-25K19.1, KIAA0125, MAGI2-AS3, and DLX6-AS1 are 7, 19, 32, and 36, respectively. Interestingly, KIAA0125 is found to have a tumor suppressor effect that regulates the development and metastasis of colon cancer [48]. The function of MAGI2-AS3 was verified in the correlation analysis. DLX6-AS1 is found to act as a ceRNA of miR-577 to accelerate the malignant development of colon cancer [49]. As for RP11-25K19.1, it has been found to be differentially expressed in diffuse large-B-cell lymphoma and has a good prognostic effect on the tumor [50].

### Screening of lncRNA signatures

In univariate Cox regression analysis, 30 lncRNAs were obtained by setting p value less than 0.05 (See Supplementary Table S2, Additional File 1). In RSF analysis, 13 lncRNAs were obtained by screening the lncRNAs with a score greater than or equal to 9 (See Supplementary Table S3, Additional File 1). Lasso regression analysis was performed after taking a union of the results of univariate Cox analysis and RSF analysis. Specifically, 34 lncRNAs were used as input for lasso regression analysis, and 14 lncRNAs with lasso regression coefficients were obtained (Fig. 3c and d). Finally, 14 lncRNAs were preliminarily screened through the above three regression analyses.

There were 379 mRNAs and 68 lncRNAs obtained when we set *α*=0.7 in the co-regulatory network (the order of matrix *C*_*ML*_(0.7) was 379∗68). There were 65 mRNAs related to colon cancer in the cosmic database. By comparing with these 65 mRNAs, RSPO3 (ENSG00000146374.12) and SFRP4 (ENSG00000106483.10) in matrix *C*_*ML*_(0.7) were found to be related to the occurrence of colon cancer. More importantly, 5 lncRNAs (ENSG00000237125.7, ENSG00000166770.9, ENSG00000227051.5, ENSG000-00234456.6, and ENSG00000255248.5) were found to be related to these two mRNAs. Subsequently, multivariate Cox analysis was fulfilled by taking the union of the lncRNAs obtained from lasso analysis and these 5 lncRNAs. A total of 19 lncRNAs were used for multivariate Cox analysis. Three lncRNAs with high p values were deleted, and 16 lncRNAs were left for the final analysis. Six lncRNAs were found to be significantly correlated with the overall survival of colon cancer samples (*p* <0.05), and the univariate and multivariate Cox analysis results of these lncRNAs are shown in Table 3 (ENSG00000166770.9 comes from correlation analysis).

**Table 3:** Univariate and multivariate Cox analysis

Ensembl ID	Univariate		Multivariate		
	HR (95% CI for HR)	*p* value	Coef	*z*	*p*
ENSG00000259347.4	1 (1–1)	0.0032	0.0126948	3.341	0.000833
ENSG00000228437.4	1 (1–1)	1.40E −05	0.0011064	3.045	0.002331
ENSG00000253405.1	1 (1–1)	3.30E −05	0.0018182	2.907	0.003647
ENSG00000271797.1	0.97 (0.95–0.99)	0.0018	− 0.0342018	− 2.291	0.021966
ENSG00000166770.9	–	–	0.0061149	2.087	0.03691
ENSG00000264016.2	0.97 (0.95–0.99)	0.0072	− 0.0299009	− 2	0.045526

### Model analysis and validation

**Fig. 7:**
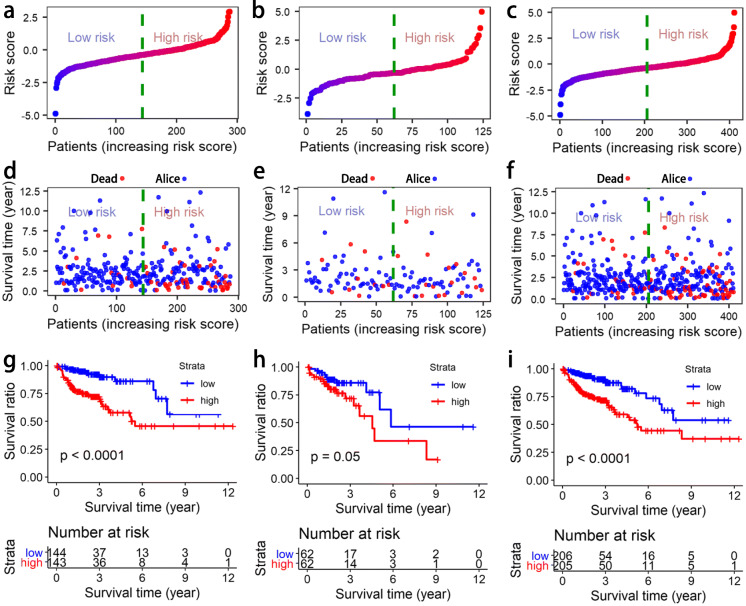
The risk score distribution, sample survival time, and Kaplan-Meier (KM) curve in the training, testing, and total set. **a** The risk score distribution in the training set. **b** The risk score distribution in the testing set. **c** The risk score distribution in the total set. **d** The sample survival time in the training set. **e** The sample survival time in the testing set. **f** The sample survival time in the total set. **g** The KM curve in the training set. **h** The KM curve in the testing set. **i** The KM curve in the total set

The six lncRNAs in Table 3 were subjected to survival analysis in the training, testing, and total set (See Supplementary Table S1, Additional File 1). The risk scores of the samples in these three sets were calculated as follows: risk score = (0.0126948 × expression level of ENSG00000259347.4) + (0.0011064 × expression level of ENSG00000228437.4) + (0.0018182 × expression level of ENSG00000253405.1) + (− 0.0342018 × expression level of ENSG00000271797.1)+ (0.0061149 × expression level of ENSG00000166770.9) + (− 0.0299009 × expression level of ENSG00000264016.2). We first analyzed the distribution of risk scores and the relationship between risk level and overall survival (Fig. 7a–f). From the scatter plot (Fig. 7d–f), it is found that the risk level can significantly fit the overall survival of colon cancer patients in the training, testing, and total set. Then, three groups of Kaplan-Meier (KM) survival curves were constructed, as shown in Fig. 7g–i. It can be found that these six lncRNAs can clearly distinguish the high and low levels of the survival rate.

**Fig. 8:**
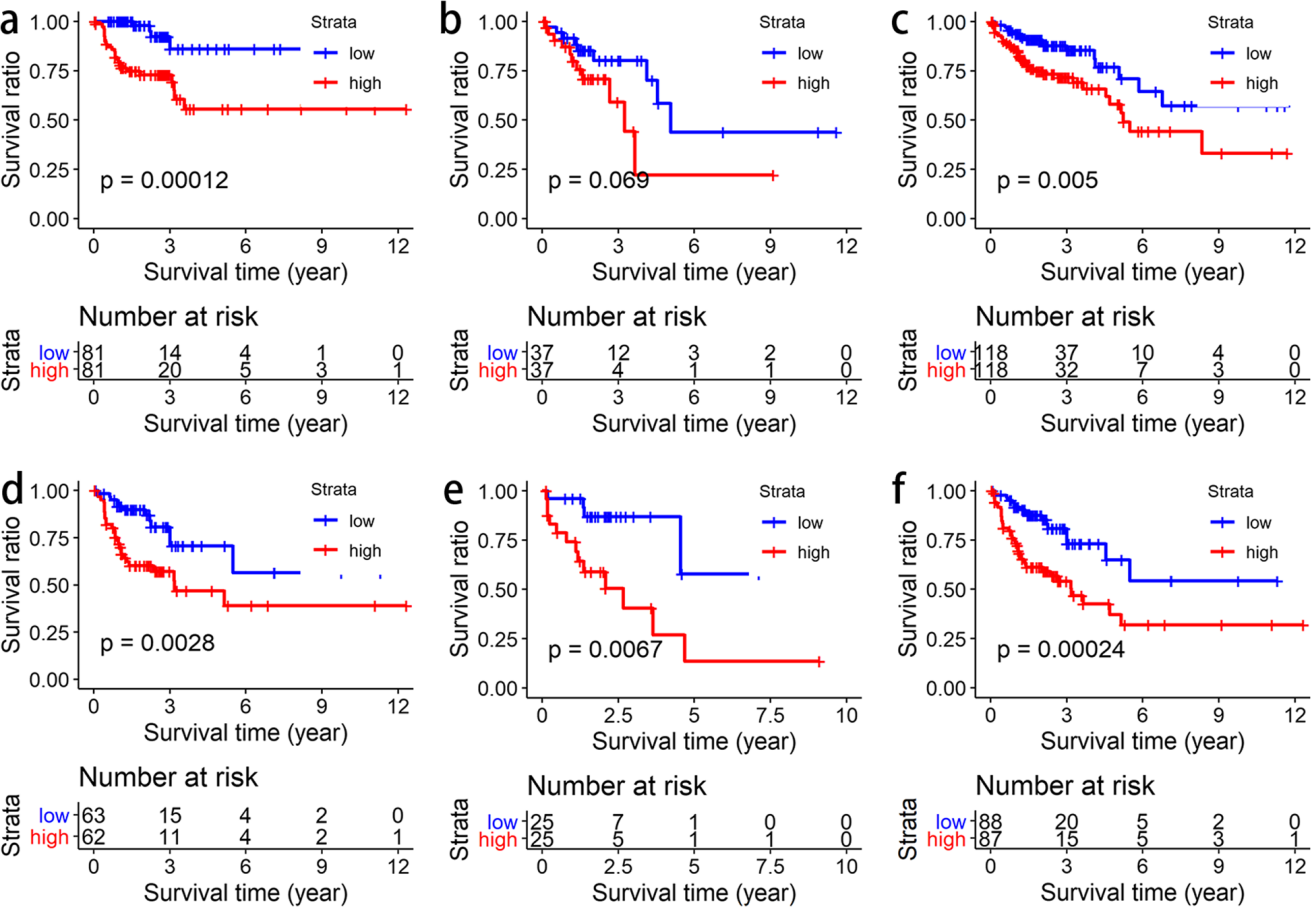
The Kaplan-Meier (KM) curves of early-stage (I/II) and late-stage (III/IV) samples. **a** The KM curve of early-stage samples in the training set. **b** The KM curve of early-stage samples in the testing set. **c** The KM curve of early-stage samples in the total set. **d** The KM curve of late-stage samples in the training set. **e** The KM curve of late-stage samples in the testing set. **f** The KM curve of late-stage samples in the total set

In order to further analyze and validate our prognostic model, we obtained six sample sets (early-stage samples in the training set, late-stage samples in the training set, early-stage samples in the testing set, late-stage samples in the testing set, early-stage samples in the total set, and late-stage samples in the total set) through collecting the colon cancer samples by their stages. Among them, samples from stage I/II belong to the early-stage group and samples from stage III/IV belong to the late-stage group. Then, we performed survival analysis on these six sets (Fig. 8). The results show that our model has good prognostic performance in both the early-stage and late-stage groups. We also analyzed the risk score distribution and overall survival of the samples in these 6 sets (See Supplementary Figure S1, Additional File 1). We found that samples with high risk levels were more likely to die than those with low risk levels in these sets, which is consistent with the expected results.

In summary, these six lncRNA signatures can significantly fit the overall survival of the sample, and the prognostic model composed of them can provide an effective prognosis for patients with colon cancer.

### Independence of the prognostic model

In order to analyze the relationship between the the prognostic signatures of lncRNA and other clinical factors, we performed univariate and multivariate Cox regression analysis on the risk score and 6 other clinical characteristics (age, gender, tumor stage, tumor invasion, lymph node, and metastasis) (Table 4). We found that in the three sets, only the risk score <= 0.05 in both univariate and multivariate Cox analysis. This indicates that the six lncRNAs we identified are independent prognostic factors for colon cancer patients, that is, our prognostic model can predict the overall survival of colon cancer patients independently of other clinically relevant characteristics.

**Table 4:** Univariate and multivariate Cox analysis of clinical characteristics on three sets

Variables	Univariate		Multivariate		
	HR (95% CI for HR)	*p* value	Coef	*z*	*p*
**Training set**					
Age	1 (1–1.1)	0.016	0.0442637	3.290	0.0010
Gender	0.92 (0.54–1.6)	0.75	0.0004572	0.002	0.9987
Tumor stage	2.2 (1.6–3)	3.3e −07	0.4570372	1.739	0.0820
Tumor invasion (T)	2.9 (1.8–4.8)	1.8e-05	0.6198692	1.871	0.0614
Lymph node (N)	2.2 (1.6–2.9)	9.5e −07	0.2121219	0.860	0.3895
Metastasis (M)	1.7 (1.2–2.3)	0.001	0.2204375	1.065	0.2869
Risk score	2 (1.6–2.6)	**6.4e −08**	0.6528934	4.518	**6.24e −06**
**Testing set**					
Age	1 (0.98–1)	0.82	0.02209	1.359	0.17421
Gender	0.76 (0.36–1.6)	0.49	−0.19816	−0.454	0.65005
Tumor stage	2.8 (1.7–4.7)	6.2e −05	0.94479	2.720	0.00653
Tumor invasion (T)	1.8 (0.86–3.8)	0.12	−0.10185	−0.203	0.83914
Lymph node (N)	2.1 (1.3–3.4)	0.0034	−0.10574	−0.287	0.77424
Metastasis (M)	1.6 (1–2.5)	0.031	0.51952	1.630	0.10307
Risk score	1.4 (1–1.8)	**0.027**	0.34615	2.071	**0.03837**
**Total set**					
Age	1 (1–1)	0.041	0.03938	3.833	0.000127
Gender	0.89 (0.58–1.4)	0.6	−0.02656	−0.115	0.908684
Tumor stage	2.3 (1.8–2.9)	1.5e −10	0.59692	2.802	0.005076
Tumor invasion (T)	2.6 (1.7–4)	5e −06	0.45556	1.691	0.090824
Lymph node (N)	2.1 (1.6–2.7)	2.1e −08	0.08203	0.394	0.693225
Metastasis (M)	1.6 (1.3–2.1)	0.00012	0.31324	1.870	0.061471
Risk score	1.6 (1.4–2)	**2.8e −08**	0.49875	4.845	**1.27e −06**

## Discussion

Studies have shown that abnormal transcription of lncRNA is related to the occurrence of colon cancer [11, 12, 14]. LncRNA has become a promising prognostic biomarker candidate for colon cancer. It is necessary to find significant lncRNA signatures to predict the overall survival of colon cancer patients. In this study, we conducted a comprehensive analysis of secondary structure correlation fusion, construction of ceRNA regulatory network, and identification lncRNA prognostic signatures. Finally, a risk prognosis model for colon cancer samples based on 6 lncRNA signatures was proposed, which provides further insights into the prognosis of lncRNAs in colon cancer.

Four hub-lncRNAs (RP11-25K19.1, KIAA0125, MA-GI2-AS3, and DLX6-AS1) were identified in the ceRNA regulatory network. We speculate that these lncRNAs may play important regulatory roles in colon cancer. KIAA0125 has been found to have a tumor suppressor effect that regulates the development and metastasis of colon cancer [48]. As for MAGI2-AS3, it has been found to promote the progression of colon cancer by regulating the miR-3163/TMEM106B axis [46]. DLX6-AS1 has been found to act as a ceRNA of miR-577 to accelerate the malignant development of colon cancer [49]. Therefore, based on the above results, we can infer that RP11-25K19.1 also plays an important regulatory role in colon cancer, and this regulatory mechanism is achieved through the ceRNA network.

Subsequently, through gene function analysis of the target mRNAs in the co-regulated relationship, we found that these colon cancer-related mRNAs are related to GO terms such as signal transduction, integral component of membrane, and calcium ion binding. And these mRNAs are mainly enriched in the PI3K-Akt signaling pathway through KEGG pathway enrichment analysis. These enriched GO terms and KEGG pathways are related to the life cycle of colon cancer cells, and it is reported that the signal transduction, integral component of membrane, and calcium ion binding are related to cell growth, division, and death [51]. The activation of the signal transduction can lead to the occurrence of colon cancer [52]. The PI3K-Akt signaling pathway is related to the regulation of cell growth cycle, and it has been found to be mutated in cancers [53]. Besides, it has also been reported that the activity of PI3K-Akt signaling pathway is increased in colon cancer [47]. It is possible to induce apoptosis of cancer cells by studying targeted drugs related to PI3K-Akt to achieve the purpose of cancer treatment [53].

Finally, 6 lncRNAs related to the overall survival of colon cancer were found. The sources of these lncRNAs are shown in Table 5. Especially, the EVX1-AS, ZNF667-AS1, CTC-428G20.6, and CTC-297N7.9 were first found to be related to colon cancer, where the EVX1-AS and ZNF667-AS1 have been predicted to be related to colon cancer in LncRNADisease (V2.0) (the latter was verified in the correlation analysis). The RP11-798K3.2 and RP11-400N13.2 have been proven to be related to colon cancer by previous studies [34, 35]. We further explored the performance of the prognostic model on drug treatment and radiotherapy samples(See Supplementary Figure S2 and Figure S3, Additional File 1). The results show that the lncRNA signatures we found can prognosticate the survival risk of colon cancer patients independently of the type of treatment, and there is no significant difference in the overall survival of samples with different treatments. In addition, we compared the prognostic model composed of these six lncRNA features with four other models related to colon cancer (See Supplementary Table S4, Additional File 1). It can be found that only our prognostic method considers both structural information and expression value information, which is of great significance for the discovery of potential lncRNA characteristics in colon cancer.

**Table 5:** Source of lncRNAs in risk prognosis model

Ensembl ID	Gene name	Source
ENSG00000259347.4	RP11-798K3.2	PMID: 29227531
ENSG00000228437.4	RP11-400N13.2	PMID: 31516583
ENSG00000253405.1	EVX1-AS	LncRNADisease
ENSG00000166770.9	ZNF667-AS1	LncRNADisease, Correlation
ENSG00000264016.2	CTC-297N7.9	–
ENSG00000271797.1	CTC-428G20.6	–

Although our method has a good performance in the prognosis of colon cancer, it still needs to be improved from the following two aspects. One is that our prognostic model was trained based on colon cancer samples, and there is no guarantee that it can still achieve good results on other cancer data sets. The other is that we only considered the sequence information and secondary structure information of lncRNA, but other information such as tertiary structure information may also affect its expression. In future work, we plan to add more interesting information to identify prognostic-related lncRNA signature. Besides, If conditions permit, we will conduct experimental verification on the newly discovered lncRNA signatures related to colon cancer.

## Conclusions

This study identified the potential regulatory relationships between lncRNAs and mRNAs by integrating their expression values and secondary structure information. Six lncRNA signatures were found to be related to the prognosis of colon cancer, two of which were found to be associated with colon cancer for the first time. A risk prognostic model based on these six lncRNAs was proposed. This model not only helps to comprehend the mechanism of colon cancer at the long-noncoding level, but also provides a reference for the prognosis of colon cancer patients.

## Supplementary Information

**Additional file 1** Table S1. Clinical characteristics of colon cancer samples. Table S2. The results of univariate Cox analysis. Table S3. The results of Random Survival Forest analysis. Table S4 Comparative analysis with other prognostic methods. Figure S1. The risk score distribution and sample survival time of early-stage (I/II) and late-stage (III/IV) samples. Figure S2. The Kaplan-Meier (KM) curve of pharmaceutical therapy and radiation therapy samples. Figure S3. The relationship between treatment type and overall survival

## Data Availability

All required data are included in this manuscript. Declarations

## Abbreviations

lncRNA: Long non-coding RNA
COAD: Colon adenocarcinoma
ceRNA: Competing endogenous RNA
SS: Secondary structure
RSF: Random survival forest
KM: Kaplan Meier
GO: Gene Ontology
KEGG: Kyoto Encyclopedia of Genes and Genomes
