# Supplementary material for: Comprehensive analyses of correlation and survival reveal informative lncRNA prognostic signatures in colon cancer
Source: World J Surg Oncol. 2021 Apr 9;19:104. doi: 10.1186/s12957-021-02196-4 (PMC8035745; doi:10.1186/s12957-021-02196-4)
Supplement: Supplementary file 1 — Additional file 1 Table S1. Clinical characteristics of colon cancer samples. Table S2. The results of univariate Cox analysis. Table S3. The results of Random Survival Forest analysis. Table S4 Comparative analysis with other prognostic methods. Figure S1. The risk score distribution and sample survival time of early-stage (I/II) and late-stage (III/IV) samples. Figure S2. The Kaplan-Meier (KM) curve of pharmaceutical therapy and radiation therapy samples. Figure S3. The relationship between treatment type and overall survival [file 12957_2021_2196_MOESM1_ESM.docx]

Supplementary data

Table S1 Clinical characteristics of colon cancer samples.

| Variables | | Total (n=411) | Training (n=287) | Testing (n=124) |
| --- | --- | --- | --- | --- |
| Age | <=65 | 171 | 116 | 55 |
|  | >65 | 240 | 171 | 69 |
| Gender | male | 218 | 154 | 64 |
|  | female | 193 | 133 | 60 |
| Survival status | Dead | 327 | 230 | 97 |
|  | Alive | 84 | 57 | 27 |
| Tumor stage | I | 73 | 50 | 23 |
|  | II | 163 | 112 | 51 |
|  | III | 117 | 83 | 34 |
|  | IV | 58 | 42 | 16 |
| Tumor invasion (T) | T1 | 9 | 6 | 3 |
|  | T2 | 71 | 48 | 23 |
|  | T3 | 282 | 198 | 84 |
|  | T4 | 48 | 34 | 14 |
|  | unknown | 1 | 1 | 0 |
| Lymph node (N) | N0 | 244 | 166 | 78 |
|  | N1 | 96 | 68 | 28 |
|  | N2 | 71 | 53 | 18 |
| Metastasis (M) | M0 | 311 | 218 | 93 |
|  | M1 | 58 | 42 | 16 |
|  | MX | 39 | 25 | 14 |
|  | unknown | 3 | 2 | 1 |

Table S2 The results of univariate Cox analysis.

| Ensembl ID | beta | HR(95% CI for HR) | wald.test | p.value |
| --- | --- | --- | --- | --- |
| ENSG00000228437.4 | 0.001 | 1 (1-1) | 19 | 1.40E-05 |
| ENSG00000253405.1 | 0.0014 | 1 (1-1) | 17 | 3.30E-05 |
| ENSG00000255571.5 | 0.0058 | 1 (1-1) | 16 | 6.10E-05 |
| ENSG00000245526.7 | 0.023 | 1 (1-1) | 11 | 0.00079 |
| ENSG00000275216.1 | 0.00021 | 1 (1-1) | 11 | 0.00089 |
| ENSG00000163009.7 | 0.002 | 1 (1-1) | 11 | 0.00097 |
| ENSG00000271797.1 | -0.03 | 0.97 (0.95-0.99) | 9.7 | 0.0018 |
| ENSG00000230798.4 | 0.013 | 1 (1-1) | 9.8 | 0.0018 |
| ENSG00000235532.1 | 0.011 | 1 (1-1) | 9.7 | 0.0019 |
| ENSG00000225335.3 | -0.0057 | 0.99 (0.99-1) | 9 | 0.0027 |
| ENSG00000259347.4 | 0.0097 | 1 (1-1) | 8.7 | 0.0032 |
| ENSG00000205056.8 | 0.0063 | 1 (1-1) | 7.3 | 0.0067 |
| ENSG00000264016.2 | -0.028 | 0.97 (0.95-0.99) | 7.2 | 0.0072 |
| ENSG00000268505.1 | -0.012 | 0.99 (0.98-1) | 6.5 | 0.011 |
| ENSG00000268388.4 | -0.00046 | 1 (1-1) | 6.4 | 0.012 |
| ENSG00000265485.4 | -0.01 | 0.99 (0.98-1) | 6.3 | 0.012 |
| ENSG00000225383.5 | 0.0086 | 1 (1-1) | 5.9 | 0.015 |
| ENSG00000214888.2 | 0.024 | 1 (1-1) | 5.3 | 0.021 |
| ENSG00000236333.3 | 0.0072 | 1 (1-1) | 5.1 | 0.023 |
| ENSG00000237070.1 | 0.00043 | 1 (1-1) | 5.2 | 0.023 |
| ENSG00000180869.4 | -0.0034 | 1 (0.99-1) | 5.2 | 0.023 |
| ENSG00000265356.1 | 0.039 | 1 (1-1.1) | 5 | 0.025 |
| ENSG00000267480.1 | -0.0049 | 1 (0.99-1) | 5 | 0.026 |
| ENSG00000235884.3 | 6e-04 | 1 (1-1) | 4.8 | 0.028 |
| ENSG00000218416.4 | 0.002 | 1 (1-1) | 4.7 | 0.031 |
| ENSG00000254973.1 | 0.0033 | 1 (1-1) | 4.5 | 0.034 |
| ENSG00000260877.1 | 0.0021 | 1 (1-1) | 4.5 | 0.034 |
| ENSG00000240498.5 | -0.0014 | 1 (1-1) | 4.1 | 0.043 |
| ENSG00000231412.2 | 0.001 | 1 (1-1) | 4 | 0.045 |
| ENSG00000246334.2 | 0.0032 | 1 (1-1) | 3.9 | 0.049 |

Table S3 The results of Random Survival Forest analysis.

| Ensembl ID | rel.freq |
| --- | --- |
| ENSG00000228437.4 | 16 |
| ENSG00000233554.4 | 13 |
| ENSG00000237438.5 | 13 |
| ENSG00000230798.4 | 12 |
| ENSG00000246334.2 | 11 |
| ENSG00000163009.7 | 11 |
| ENSG00000268388.4 | 10 |
| ENSG00000267530.2 | 10 |
| ENSG00000259347.4 | 10 |
| ENSG00000223573.5 | 9 |
| ENSG00000267480.1 | 9 |
| ENSG00000265485.4 | 9 |
| ENSG00000253405.1 | 9 |

Table S4 Comparative analysis with other prognostic methods.

| Mthhod | PubMed | LncRNAs related to prognosis (no) | The information involved |
| --- | --- | --- | --- |
| Mthhod1 | 31448228 | 5 | Expressed value information |
| Mthhod2 | 31824849 | 3 | Expressed value information |
| Mthhod3 | 29227531 | 6 | Expressed value information |
| Mthhod4 | 31516583 | 2 | Expressed value information |
| Our method | - | 6 | Expressed value information,  secondary structure information |


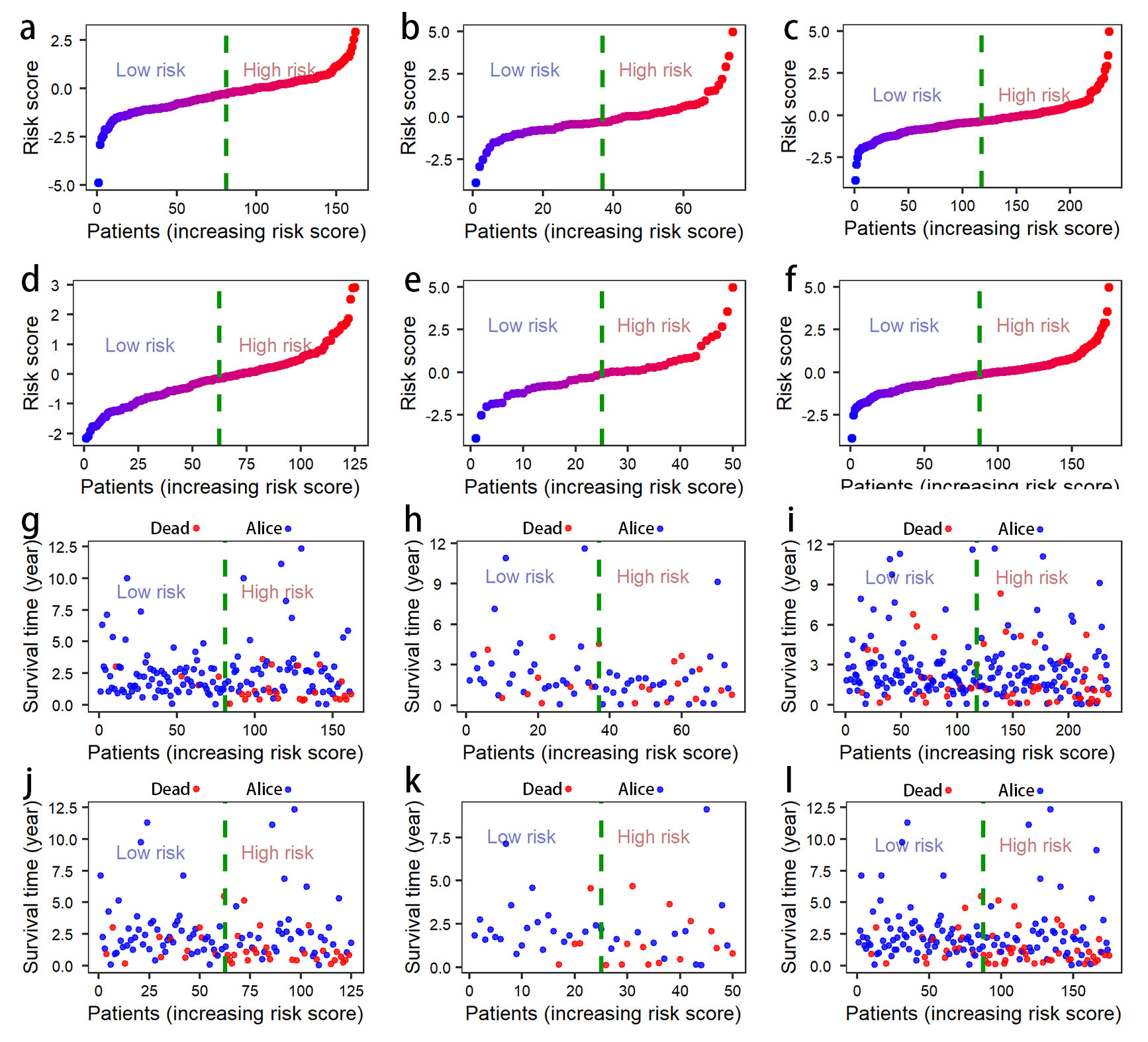


Figure S1 The risk score distribution and sample survival time of early-stage (I/II) and late-stage (III/IV) samples. (a) The risk score distribution of early-stage samples in the training set. (b) The risk score distribution of early-stage samples in the testing set. (c) The risk score distribution of early-stage samples in the total set. (d) The risk score distribution of late-stage samples in the training set. (e) The risk score distribution of late-stage samples in the testing set. (f) The risk score distribution of late-stage samples in the total set. (g) The overall survival of early-stage samples in the training set. (h) The overall survival of early-stage samples in the testing set. (i) The overall survival of early-stage samples in the total set. (j) The overall survival of late-stage samples in the training set. (k) The overall survival of late-stage samples in the testing set. (l) The overall survival of late-stage samples in the total set.


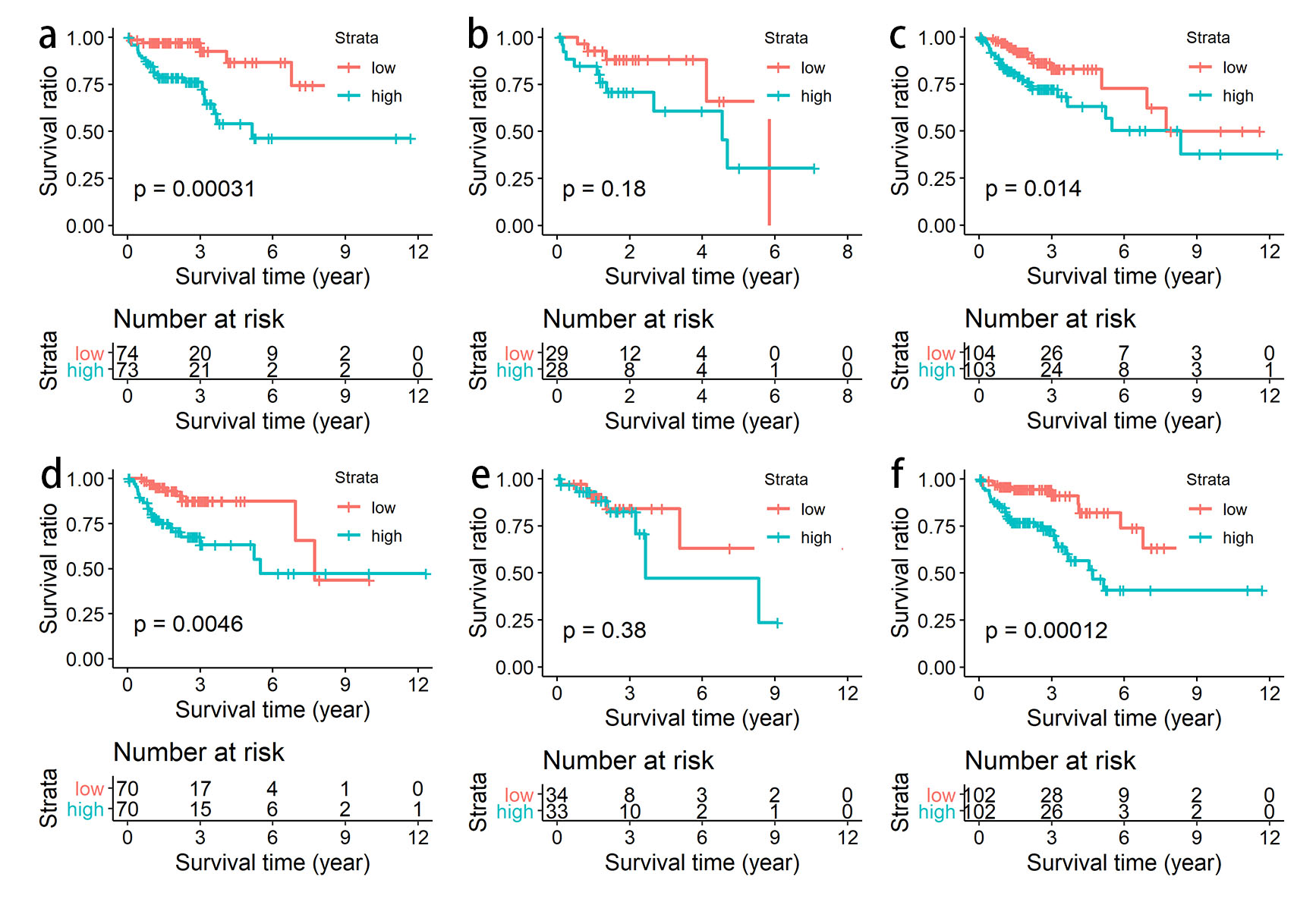


Figure S2 Kaplan-Meier (KM) curves of samples of different treatment types. (a) Pharmaceutical therapy samples in the training set. (b) Pharmaceutical therapy samples in the testing set. (c) Pharmaceutical therapy samples in the total set. (d) Radiation therapy samples in the training set. (e) Radiation therapy samples in the testing set. (f) Radiation therapy samples in the total set.


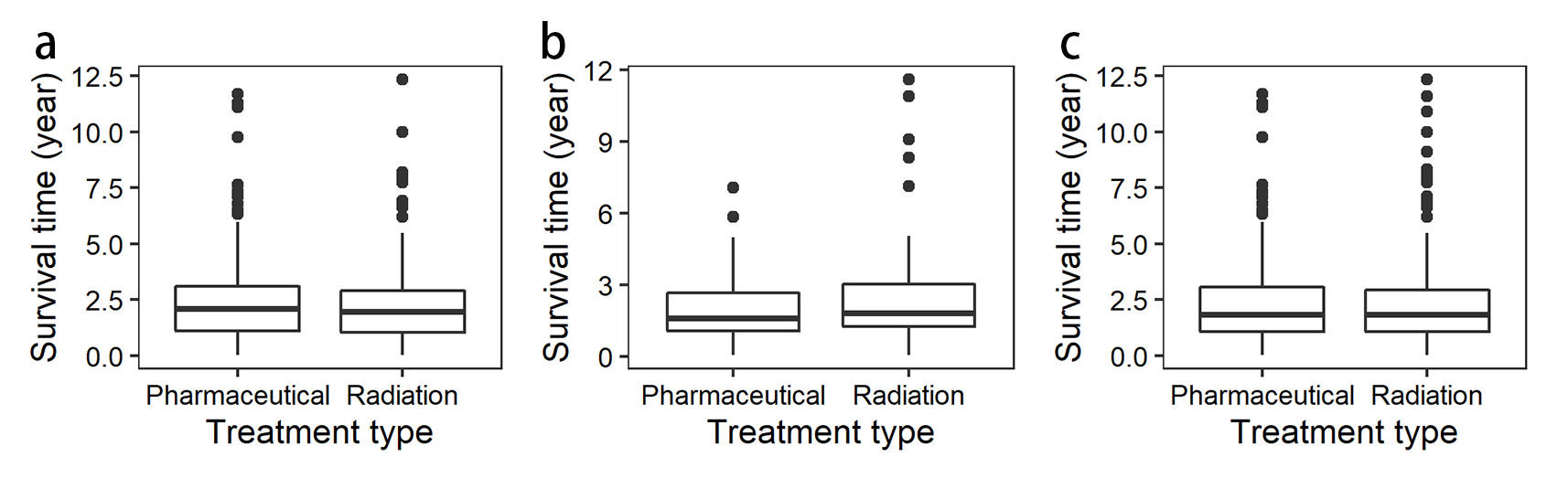


Figure S3 The relationship between treatment type and overall survival. (a) Training set. (b) Testing set. (c) Total set.
